# 2025 ACVIM Forum Research Report Program

**DOI:** 10.1111/jvim.70255

**Published:** 2025-09-30

The American College of Veterinary Internal Medicine (ACVIM) Forum and the Journal of Veterinary Internal Medicine (JVIM) are not responsible for the content or dosage recommendations in the abstracts. The abstracts are not peer reviewed before publication. The opinions expressed in the abstracts are those of the author(s) and may not represent the views or position of the ACVIM. The authors are solely responsible for the content of the abstracts.

**2025 ACVIM Forum**

**June 18 to October 31, 2025**

**Research Report Program**

**Index of Abstracts**

**Table:** 

**Presenter**	**Title**
**CARDIOLOGY**	
Ta‐Li Lu	Evolution of Plasma NT‐proBNP Concentrations and Echocardiographic Changes in Cats Followed for ≥ 5 Years
Simone Cupido	Complications and Outcome in 306 Dogs Undergoing Echo‐guided Interventional Cardiac Procedures
Jacob Dunham	Impact of Leaflet‐to‐Annulus Index on Residual Regurgitation in Dogs Undergoing Mitral Valve Transcatheter Edge‐to‐Edge Repair
Justin Ringhofer	Efficacy of Pre‐emptive Lidocaine for Prevention of Ventricular Arrhythmias in Dogs Undergoing Balloon Pulmonary Valvuloplasty
Sorawit Phetariyawong	Digital Histopathological Analysis of Myocardial Tissue in Canine Myxomatous Mitral Valve Disease and Dilated Cardiomyopathy
Ilana Levinzon	Prevalence of Novel Mutations and Association with Dilated Cardiomyopathy in North American Doberman Pinschers
**EQUINE**	
Gunther Van Loon	Right Atrial Free‐Wall Premature Depolarizations in Horses and Experience with 3D‐Electro‐Anatomical Mapping (Carto) and Ablation
Lutz Goehring	How Much Air‐borne Molecular Equid Alphaherpesvirus 1 is at (International) Competitions? Estimating “the Cloud”
Megan Palmisano	Retrospective Analysis of Multisite versus Single Site Blood Cultures in Neonatal Foals
Rachel Pfeifle	Effects of Nebulized Alpha‐2‐Macroglobulin in Adult, Asthmatic Horses
Barbara Delvescovo	Pharmacokinetics of Oral Ursodeoxycholic Acid and its Impact on Bile Acid Profiles in Horses
**FOOD ANIMAL INTERNAL MEDICINE**	
Lisa Gamsjäger	Impact of Three Colostrum Replacement Strategies on Immunoglobulin G Absorption and Growth in Beef Calves
Jennifer Halleran	Pharmacokinetics of Phenazopyridine in Healthy Goats at Two Different Dosing Regimens
Luiza Zakia	Assessing the Effectiveness of an Evidence‐Based Algorithm for Antimicrobial Treatment in Neonatal Calf Diarrhea
**NEUROLOGY**	
Rell Parker	Questionnaire Based Study of the Correlation Between Sleep and Pain in Cavalier King Charles Spaniels
Adrien Dupanloup	Outcome, Complications and Risk Factors for Perioperative Mortality in Dogs Undergoing Caudal Fossa Surgery
Han Sun	Prazosin versus Tamsulosin for Urinary Retention in Non‐ambulatory Dogs Following Surgically Treated Intervertebral Disc Herniation
Sheila Carrera‐Justiz	Veterinary Neurologists' Approach to French Bulldogs with Spinal Cord Disease and Brachycephalic Obstructive Airway Syndrome
**NUTRITION**	
Sally Perea	Highly Hydrolyzed Versus Novel Protein Diet in the Management of Canine Chronic Inflammatory Enteropathy
**ONCOLOGY**	
Doyun Kim	Clinical Efficacy and Tolerability of Lapatinib in Metastatic Canine Mammary Carcinomas: A Multi‐Center Pilot Study
Chick Weisse	Prognosis and Associated Risk Factors Following Conservative Management of Dogs with Large Liver Tumors
**SMALL ANIMAL INTERNAL MEDICINE**	
Chick Weisse	Outcomes and Long‐Term Survival in Animals Treated for Hepatic Arteriovenous Malformations (HAVM)
Anne Kimmerlein	Prolonged Antibody Responses in Dogs and Cats Exposed to COVID‐19 Diagnosed Pet Owners
Marion Allano	Infection Prevention and Control Programs at Avma‐accredited Veterinary Teaching Hospitals
Autumn Harris	Relationship Between Urinary Ammonia Excretion and Survival Time in Dogs with Kidney Disease
Elizabeth Rozanski	Prevalence of B‐lines in Cats with Asthma
Audrey Cook	Demographics, Imaging Findings, Diagnosis, and Outcome for 74 Dogs Undergoing Adrenalectomy for an Incidental Mass
Oliver Waite	Risk Factors Against Treating Cats with Diabetes Mellitus and ≤ 30‐day Survival After Starting Anti‐Hyperglycaemic Therapy
Christian Leutenegger	Comparative Evaluation of Two Canine Vector‐Borne Disease Pathogen PCR Panels
Sue Yee Lim	Clinical Experience With Fuzapladib Sodium Treatment in Dogs with Complicated Acute Pancreatitis
Linda Toresson	Fecal Microbiota Transplantation as Adjunct Treatment in Dogs with Refractory CE: A Prospective Study
Linda Toresson	Treatment with Bile Acid Sequestrants in Dogs with Refractory Chronic Enteropathies
Kevin Murtagh	Neutrophilic Inflammatory Enteropathy in Dogs: A Retrospective Descriptive Study
Kevin Murtagh	Insect‐based Novel Protein Diet for Dogs with Chronic Enteropathy: A Prospective Study
Meg Nakazawa	Evaluating the Impact of Preparation Conditions on Bacterial Viability in Canine Fecal Microbiota Transplant Capsules
Alison Manchester	Transcriptomic Interrogation of the Duodenal Mucosa of Dogs with Chronic Enteropathy

## 1 CARDIOLOGY

## 2 Complications and Outcome in 306 Dogs Undergoing Echo‐Guided Interventional Cardiac Procedures

### 2.1 **Simone Cupido** ^1^; Federica Valeri^2^; Francesco Birettoni^2^; Domenico Caivano^2^; Alessandro Fruganti^3^; Patrizia Knafelz^4^; Giulia Costa^4^; Francesco Porciello^2^

#### 2.1.1 ^1^PhD Student, University of Perugia; ^2^Veterinary Medicine, University of Perugia; ^3^Veterinary Medicine, University of Camerino; ^4^Gregorio VII Veterinary Hospital

**BACKGROUND:** Minimally invasive interventional procedures are routinely performed in veterinary cardiology. Fluoroscopy is typically used for procedure guidance, aside from ultrasound guidance. Common therapeutic indications for interventional procedures include closure of patent ductus arteriosus (PDA), pulmonary balloon valvuloplasty (PBV), and permanent artificial pacemaker implantation (PMI).

**OBJECTIVE:** Short‐term outcomes and complications of PDA occlusion, PBV, and PMI using only ultrasound guidance have never been described before. This study aims to report short‐term outcomes and complications in three referral centers between 2009 and 2024.

**ANIMALS:** Three hundred and six client‐owned dogs were presented for PDA occlusion, PBV, or PMI, 130 (91 females, 39 males), 144 (59 females, 85 males), and 32 (18 females, 14 males), respectively.

**RESULTS:** In the PDA group, 4 dogs (3.4%) had intra‐procedure complications, divided into major (1.7%) and minor (1.7%). Twenty‐one dogs (17.95%) had short‐term post‐procedural complications, divided into major (1.74%) and minor (16.67%). In the PS group, 20 dogs (16%) had intra‐operatory complications, divided into major (7.2%) and minor complications (8.8%). One dog (0.8%) had major short‐term post‐procedural complications. In the PMI group, 2 dogs (6.2%) had complications during the procedure, whereas 5 dogs (15.6%) had post‐implantation complications. The short‐term survival rate in the PDA occlusion, PBV, and PMI groups was 100%, 98.4%, and 93.7%, respectively.

**CONCLUSIONS:** The complication outcome rate is similar to what is reported for the same procedures performed under fluoroscopic guidance in other veterinary institutions. Our study demonstrates that ultrasound guidance in interventional cardiology procedures is safe and effective in PDA occlusion, PBV, and PMI.

## 3 Impact of Leaflet‐to‐Annulus Index on Residual Regurgitation in Dogs Undergoing Mitral Valve Transcatheter Edge‐to‐Edge Repair

### 3.1 **Jacob Dunham** ^1^; Saki Kadotani^2^, DVM, DACVIM (Cardiology); Carl Toborowsky^2^, VMD, DACVIM (Cardiology); Emily Javery^2^, DVM; Sumana Prabhakar^2^, DVM; Tod Sumerfield^2^; DVM; Ryan Fries^2^, DVM, DACVIM (Cardiology), DECVIM‐SA (Cardiology)

#### 3.1.1 ^1^Small Animal Rotating Intern, University of Illinois Urbana‐Champaign; ^2^University of Illinois

**BACKGROUND:** Mitral valve transcatheter edge‐to‐edge repair (TEER) can reduce the severity of mitral regurgitation (MR) and improve prognosis for dogs with myxomatous mitral valve disease. Leaflet‐to‐annulus index (LAI), a measure of leaflet annular mismatch, may impact procedural success.

**HYPOTHESIS/OBJECTIVES:** We hypothesized that LAI will significantly impact 24‐h postoperative regurgitant fraction (RF) in dogs undergoing TEER.

**ANIMALS:** Exactly 43 client‐owned dogs (28 stage C, 15 stage B2) undergoing mitral valve TEER

**METHODS:** Prospective observational study. The following variables were evaluated for their impact on procedural success: LAI, operator experience, commissural vena contracta width (VC%), device to annulus ratio, and anterior‐posterior dimension (AP_diam_). Exactly 24‐h postoperative RF was quantified using the transthoracic volumetric method. Normality was assessed, and Mann–Whitney tests were used to compare groups. Outcome variables were assessed using linear regression.

**RESULTS:** Thirty‐nine of 43 dogs (91%) survived and had a 24‐h postoperative echocardiogram. The median measurement for all dogs was: LAI = 1.0 (min 0.898, max 1.42), postoperative RF = 39% (min 12%, max 68%), VC% = 57% (min 33%, max 78%), AP dimension = 17.5 mm (min 14 mm, max 24 mm), and device to annulus ratio = 0.91 (min 0.81, max 1.06). Median operator experience was 9 months (min 1 month, max 28 months). Multiple linear regression found LAI (*p* = 0.015) and experience (*p* = 0.012) to be independent predictors of postoperative RF. Dogs with RF < 40% (*n* = 23) had significantly larger LAI (*p* = 0.02) compared with dogs with RF > 40% (*n* = 16).

**CONCLUSIONS AND CLINICAL IMPORTANCE:** LAI and operator experience significantly impact short‐term procedural success.

## 4 Prevalence of Novel Mutations and Association With Dilated Cardiomyopathy in North American Doberman Pinschers

### 4.1 **Ilana Levinzon** ^1^, DVM; Eduardo J. Benjamin^2^, DVM, MS, DACVIM (Cardiology); Oscar Hernández Maldonado^3^, BS, MS; Giulio Menciotti^4^, DVM, MS, PhD, DACVIM (Cardiology), DECVIM‐CA (Cardiology); Sarah Bell^5^, DVM, MS, DACVIM (Cardiology); Amara H. Estrada^6^, DVM, DACVIM (Cardiology)

#### 4.1.1 ^1^Cardiology Research Intern, Department of Small Animal Clinical Sciences, College of Veterinary Medicine, University of Florida; ^2^Clinical Assistant Professor of Cardiology, Department of Small Animal Clinical Sciences, College of Veterinary Medicine, University of Florida; ^3^Biological Scientist II, Clinical Sciences Research Lab, College of Veterinary Medicine, University of Florida; ^4^Assistant Professor of Cardiology, Department of Small Animal Clinical Sciences, Virginia‐Maryland College of Veterinary Medicine; ^5^CVCA Cardiac Care for Pets; ^6^Professor of Cardiology, Department of Small Animal Clinical Sciences, College of Veterinary Medicine, University of Florida

**BACKGROUND:** Doberman pinschers (DPs) are substantially affected by dilated cardiomyopathy (DCM), with a high prevalence of dogs developing systolic dysfunction and arrhythmias. While two novel mutations in RNF207 (DCM3) and PRKAA2 (DCM4) were identified in European DPs, their prevalence and association with DCM in North American populations remain unexplored.

**HYPOTHESIS/OBJECTIVES:** To determine the prevalence and association of DCM3 and DCM4 mutations with DCM in DPs in North America. We hypothesize that these mutations are not highly prevalent or associated with DCM in DPs.

**ANIMALS:** Exactly 58 DPs (55% female, 45% male), median age 6.6 years (0.1–14.1), recruited through screening clinics and dog shows.

**METHODS:** A cross‐sectional study evaluating DCM3 and DCM4 mutation status using buccal swabs in North American DPs. Cardiac evaluation included a subset of dogs (*n* = 38) using echocardiography (87%) and 24‐h Holter monitoring (58%). DCM was diagnosed using established criteria.

**RESULTS:** Of 58 DPs genotyped, 29% were positive for PDK4 mutation (DCM1; 82% heterozygous, 18% homozygous), 76% for TTN mutation (DCM2; 60% heterozygous, 40% homozygous), 87% for DCM3 (41% heterozygous, 59% homozygous), and 64% for DCM4 (78% heterozygous, 22% homozygous). DCM was diagnosed in 11% of the evaluated dogs. One was homozygous for DCM3/DCM4, one was heterozygous for DCM1/DCM2/DCM3, one was homozygous for DCM2 and heterozygous for DCM3/DCM4, and one was heterozygous for DCM3/DCM4.

**CONCLUSIONS AND CLINICAL IMPORTANCE:** DCM3 and DCM4 mutations are highly prevalent in North American Dobermans. All DCM‐affected dogs carried the DCM3 mutation. Future studies are needed to validate these associations.

## 5 Evolution of Plasma NT‐proBNP Concentrations and Echocardiographic Changes in Cats Followed for ≥ 5 Years

### 5.1 **Ta‐Li Lu** ^1^; Etienne Côté^2^, DVM, DACVIM (Cardiology, SAIM), FACC, FCAHS, FASE; Ching‐An Chen^3^, DVM, MS

#### 5.1.1 ^1^Veterinarian, Superintendent, Chuan Animal Hospital; ^2^Professor, Department of Companion Animals, Atlantic Veterinary College, University of Prince Edward Island; ^3^Veterinarian, Chuan Animal Hospital

**BACKGROUND:** Long‐term longitudinal investigations of relationships between feline [NT‐proBNP] and cardiac disease could be informative but are not reported.

**OBJECTIVES:** Evaluate associations between feline plasma [NT‐proBNP], echocardiographic findings, and time. Use baseline results to predict the development of cardiomyopathy (CM) in cats.

**METHODS:** Sixty‐three cats underwent echocardiography and measurement of plasma [NT‐proBNP] twice: at baseline and ≥ 5 years later. Correlations between [NT‐proBNP], echocardiographic findings, and time were investigated. Regression analyses sought to identify predictors of the development of CM.

**RESULTS:** Of 44 cats with normal baseline echocardiograms, 37 remained normal and 7 developed CM. Baseline [NT‐proBNP] did not differ significantly between cats that developed CM and those that remained normal. Median [NT‐proBNP] increased by 305 pmol/L in 7 cats that developed CM, versus 7 pmol in the other 37 cats. Of 57 cats with baseline [NT‐proBNP] < 100 pmol/L, 11 had [NT‐proBNP] ≥ 100 pmol/L at the second exam; 6 had developed CM. Fifty‐nine cats had negative baseline point‐of‐care (POC) NT‐proBNP; of these, 7 had developed CM on the second exam and all 7 had positive POC NT‐proBNP. Only 1/63 cats had baseline [NT‐proBNP] ≥ 100 pmol/L + normal echo, then [NT‐proBNP] ≥ 100 pmol/L + CM at the second exam. Both baseline [NT‐proBNP] and the amount it increased were significantly associated with CM.

**CONCLUSION:** Longitudinal assessments of [NT‐proBNP] and echocardiography provide insights into the emergence of feline CM. Baseline [NT‐proBNP] and the amount of change in [NT‐proBNP] are associated with CM.

## 6 Digital Histopathological Analysis of Myocardial Tissue in Canine Myxomatous Mitral Valve Disease and Dilated Cardiomyopathy

### 6.1 **Sorawit Phetariyawong** ^1^; Jens Häggström^2^; Frank G. van Steenbeek^3^; Fredrik Södersten^4^; Åsa Olsson^4^; Erik Axelsson^5^; Ingrid Ljungvall^2^

#### 6.1.1 ^1^PhD student, Swedish University of Agricultural Sciences; ^2^Clinical Sciences, Swedish University of Agricultural Sciences; ^3^Clinical Sciences, Utrecht University; ^4^Animal Biosciences, Swedish University of Agricultural Sciences; ^5^Swedish Board of Agriculture

**BACKGROUND:** Histopathological changes in cardiac tissues are incompletely evaluated in dogs with myxomatous mitral valve disease (MMVD) and dilated cardiomyopathy (DCM).

**OBJECTIVES:** Compare proportions of cardiomyocytes, fibrosis, fat, and arterial narrowing between cardiac tissue samples from dogs with MMVD, DCM, and healthy cardiac.

**ANIMALS:** Exactly 27 dogs with MMVD, 16 with DCM, and 31 cardiac healthy controls, euthanized due to reasons unrelated to the present study, were enrolled.

**METHODS:** Proportions of cardiomyocytes, fibrosis, fat, and arterial narrowing were quantified in tissue samples from multiple specific cardiac regions in each dog using a semi‐automatic quantification pipeline.

**RESULTS:** Clinical MMVD dogs had higher fibrosis proportions in the left ventricular (LV) lateral wall and posterior papillary muscle (PPM), and left atrium (LA) and higher fat proportions in the LV PPM and interventricular septum (IVS) compared with small‐breed controls (all *p* < 0.05). Clinical DCM dogs had higher fibrosis proportions in the right atrium and LA, and preclinical DCM dogs had higher fat proportions in the right ventricular (RV) lateral wall compared with large‐breed controls (all *p* < 0.05). Preclinical DCM dogs had higher fat proportions in the RV lateral wall compared with preclinical MMVD dogs, and clinical DCM dogs had higher fibrosis proportions in the IVS compared with clinical MMVD dogs (all *p* < 0.01). Arterial narrowing increased with fibrosis proportions in MMVD dogs and DCM dogs.

**CONCLUSION AND CLINICAL IMPORTANCE:** Proportions of fibrosis and fat replacement varied between cardiac locations and with disease and disease severity, which may highlight the role of these sites in disease pathogenesis.

## 7 Efficacy of Pre‐Emptive Lidocaine for Prevention of Ventricular Arrhythmias in Dogs Undergoing Balloon Pulmonary Valvuloplasty

### 7.1 **Justin Ringhofer** ^1^; Amanda Coleman^2^, DVM, DACVIM (Cardiology)

#### 7.1.1 ^1^Small Animal Rotating Intern, Veterinary Teaching Hospital, University of Georgia; ^2^College of Veterinary Medicine, University of Georgia

**BACKGROUND:** Ventricular tachyarrhythmias are a common complication during balloon pulmonary valvuloplasty (BPV). Currently, information regarding the efficacy of the antiarrhythmic lidocaine for their prevention is limited to the results of one study.

**OBJECTIVES:** To evaluate the effect of preemptive lidocaine continuous rate infusion (CRI), compared with placebo, on the incidence and severity of ventricular tachyarrhythmias in dogs undergoing BPV.

**ANIMALS:** 24 client‐owned dogs undergoing BPV for treatment of congenital pulmonary valve stenosis

**METHODS:** In this prospective, randomized, placebo‐controlled, masked clinical study, dogs were randomized to receive lidocaine (2 mg/kg IV bolus followed by 80 μg/kg/min CRI) or an equal volume of saline placebo during BVP. A standardized anesthetic protocol was used. Surface electrocardiograms were recorded continuously during the cardiac catheterization period (CCP) and later evaluated by a single masked observer. Several indicators of ventricular ectopy frequency and severity, as well as a novel composite ventricular ectopy severity score (CVESS; range of possible values, 0–13), were compared between treatment groups using a covariate or exposure variable for CCP duration. Data are presented as mean ± SD.

**RESULTS:** Twenty‐four (13 lidocaine‐treated, 11 saline‐treated) dogs were included. No significant differences in the total number of ventricular beats per hour, maximum instantaneous heart rate during ventricular tachycardia, instances of R‐on‐T, or instances of ventricular flutter were identified. Mean CVESS was not different between lidocaine‐ and saline‐treated dogs (7.1 ± 3.7 and 6.5 ± 3.7, respectively; *p* = 0.75).

**CONCLUSIONS AND CLINICAL IMPORTANCE:** Compared with placebo, pre‐emptive lidocaine administration did not result in detectable differences in ventricular arrhythmia incidence or severity.

## 8 EQUINE

## 9 Right Atrial Free‐Wall Premature Depolarizations in Horses and Experience with 3D‐Electroanatomical Mapping (Carto®) and Ablation

### 9.1 **Gunther van Loon** ^1^; Eva Buschmann^2^, DVM; Annelies Decloedt^2^; Stijn Schauvliege^2^; Glenn Van Steenkiste^2^

#### 9.1.1 ^1^Prof. Dr., Equine Cardioteam Ghent, Ghent University; ^2^Equine Cardioteam Ghent, Ghent University

**BACKGROUND:** Atrial premature depolarizations (APDs) are a known risk factor for atrial tachycardia and atrial fibrillation, and are occasionally associated with reduced performance. A 12‐lead electrocardiogram (ECG) and vectorcardiogram (VCG) might be helpful to localize the ectopic region, but 3D electro‐anatomical mapping (3D EAM) is the method of choice. Radiofrequency ablation might be used to inactivate ectopy.

**OBJECTIVES:** Describe electrocardiograms (ECGs), vectorcardiograms (VCGs), 3D EAM, and treatment by ablation.

**ANIMALS:** Three horses with a high burden of APDs from the right atrial free wall (RAFW).

**METHODS:** Records from 3 horses were reviewed.

**RESULTS:** Horses presented between 7.000 and 15.000 APDs per 24‐h and 12‐lead ECG and VCG suggested a RAFW origin. 3D EAM in two horses confirmed the RAFW origin. In the first horse, no ablation was performed as pacing (15 mA, 2 ms) of the ectopic region resulted in right phrenic nerve stimulation. The ectopic area was repetitively explored, which resulted in reduced ectopy, probably by repeated catheter pressure. The post‐procedural ECG showed only 78 APDs per 24 h. In the second horse, ablation was performed because the equine atrial wall thickness made phrenic nerve damage unlikely. After 18 applications, atrial ectopy disappeared. A 24‐h recording 6 weeks after the procedure showed only 7 APDs, which was within normal limits.

**CONCLUSIONS:** The same RAFW region appeared as the arrhythmogenic source of a high burden of APDS. The correct localization by VCG was confirmed by 3D EAM. Treatment by radiofrequency ablation was successful and did not result in side effects.

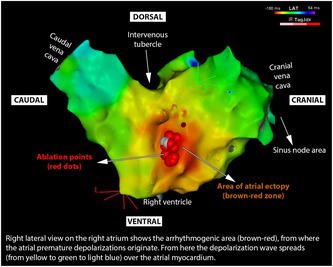

## 10 How Much Airborne Molecular Equid Alphaherpesvirus 1 is at (International) Competitions? Estimating “the Cloud”

### 10.1 **Lutz Goehring** ^1^; Eduard Jose‐Cunilleras^2^, DVM, PhD, DECEIM; Amjad Khan^3^, DVM, PhD; Edward Olajide^4^, DVM; Emma Hyde^5^

#### 10.1.1 ^1^Wright‐Markey Professor of Equine Infectious Diseases, Martin‐Gatton College of Agriculture, Food and Environment, University of Kentucky; ^2^Associate Professor, Animal Medicine and Surgery, Universitat Autonoma de Barcelona; ^3^Postdoctoral Scholar, Veterinary Science, University of Kentucky; ^4^PhD Candidate, Veterinary Science, University of Kentucky; ^5^MS Candidate, Veterinary Science, University of Kentucky

**BACKGROUND:** Equid alphaherpesvirus 1 (EHV‐1) is a respiratory tract pathogen of horses and can cause herd outbreaks of equine herpesvirusmyeloencephalopathy (EHM) or abortions. EHM outbreaks have occurred at equestrian events, and there is a need for surveillance at shows. Air space sampling during COVID‐19 has shown efficacy as a surveillance tool.

**HYPOTHESIS:** Air sampling at equestrian events will detect “airborne” EHV‐1 genome copies.

**ANIMALS:** There was no direct contact with horses.

**METHODS:** Observational study: an air sampler (Bertin™ Coriolis, France) was placed in stable units with a minimum of 5 occupied stalls out of 10. On each event day, we collected an air volume of 18 m^3^ between 10 pm and 4 am. By 9 am, we collected surface samples from each occupied stall. All samples were tested for EHV‐1, EHV‐2, and EHV‐4 using quantitative PCR and specific primers and probes. We report presence or absence in surfaces (S) or air (A) samples, as well as low (< 1000) or medium (< 10,000) abundance of target genome copies.

**RESULTS:** Ranked detection frequencies for EHV‐2, EHV‐1, and EHV‐4 were: 5 events, A16/S40; 5 events, A16/S24; and 2 events, A13/S12. Abundance was overall “low” for air or surface samples. At 2 events, we detected “medium” EHV‐1 or EHV‐4 abundance in air, which corresponded with “medium” abundance on select surfaces. EHV‐1 detection was restricted to winter events. There were no reported clinical cases during any event.

**CONCLUSIONS AND CLINICAL IMPORTANCE:** We provide evidence for the regular, low‐abundance presence of EHV‐1 and EHV‐4 genome copies at equestrian events.

## 11 Retrospective Analysis of Multisite vs. Single‐Site Blood Cultures in Neonatal Foals

### 11.1 **Megan Palmisano** ^1^; David Wong^2^; Hannah Robertson^3^; Rosemary Bayless^3^; Katarzyna Dembek^3^

#### 11.1.1 ^1^PhD Student, Department of Molecular Biomedical Sciences, North Carolina State University; ^2^Iowa State University; ^3^North Carolina State University

**BACKGROUND:** Sepsis is a detrimental disease in neonatal foals. Due to changes in hemodynamic status in critical foals, single‐site and time‐point blood culture (BC) as performed is likely to contribute to the poor yield of BC.

**HYPOTHESIS:** We hypothesize that obtaining blood cultures from multiple sites will improve identification of sepsis.

**ANIMALS:** Records from 104 foals (< 14 days old) presenting to tertiary referral centers with BC obtained on admission were reviewed.

**METHODS:** A retrospective review to compile BC results from sites collected (jugular and peripheral veins), clinical information, and sepsis scoring (SS) of the foals. Chi‐squared analysis was performed to assess BC sites collected, and a *t*‐test for analysis of SS across the populations.

**RESULTS:** Of the 104 foals, 57 were bacteremic, 59% with single‐site and 53% with two‐site BC (*p* > 0.05). Isolates varied, with the most common being *Staphylococcus* spp. (*n* = 13), *Actinobacillus* spp. (*n* = 9), *Escherichia coli* (*n* = 7), and *Bacillus* spp. (*n* = 6). Three foals with single‐site and seven foals with two‐site BC yielded only minor bacterial species often considered contaminants. The foals with minor species identified on two‐site BC had significantly greater SS of 12.7 (±2.69) compared with those with single‐site 6.7 (±3.21) (*p* = 0.015). Two of the single‐site and all seven with two‐site BC were suspected septic on clinical examination.

**CONCLUSIONS:** Larger case numbers are needed to determine whether sampling of multiple sites is a preferred method of obtaining BC to improve identification of sepsis in foals. Minor or commensal bacteria identified might be significant sources of infection or indications of sepsis.

## 12 Pharmacokinetics of Oral Ursodeoxycholic Acid and Its Impact on Bile Acid Profiles in Horses

### 12.1 **Barbara Delvescovo** ^1^; Amanda Macias^2^; Thomas Divers^2^, DVM, DACVIM, DACVECC; Callum Donnelly^1^, BVetBiol/BVetSc, DACVIM, DACT, PhD

#### 12.1.1 ^1^Assistant Professor, Cornell University; ^2^Cornell University

**BACKGROUND:** Ursodeoxycholic acid (UDCA), also known as ursodiol, is a secondary bile acid (BA) with therapeutic applications. UDCA is standard therapy for cholestatic hepatopathies in humans. In recent years, its use has been increasingly explored in equine medicine for similar indications. Pharmacokinetic data for UDCA in horses are currently lacking.

**HYPOTHESIS/OBJECTIVES:** To describe the pharmacokinetic parameters following a single intragastric administration of 15 mg/kg in healthy, fasted horses and to characterize the changes in their BA profiles.

**ANIMALS:** Nine healthy mares from Cornell research herd

**METHODS:** Horses were administered a single dose of 15 mg/kg of UDCA intragastrically. Plasma concentrations of UDCA and 19 additional bile acids were measured over 72 h using liquid chromatography‐tandem mass spectrometry (LC‐MS/MS). Pharmacokinetic data were analyzed by a non‐compartmental model.

**RESULTS:** Plasma concentrations increased rapidly, with peak concentrations (*T*_max_; mean ± SD) occurring at 2.44 ± 1.3 h. Plasma levels decreased significantly in most horses by 24 h, with negligible concentrations detected at 72 h. Half‐life was calculated at 7.99 ± 2.25 h, and the dose interval estimated at 24.29 h. The pharmacokinetic profile of taurine conjugated UDCA also supports a dose interval of 24 h. Complete BA profiles indicate transient depression of primary BA and enhancement of secondary BA following UDCA administration.

**CONCLUSIONS AND CLINICAL IMPORTANCE:** The oral dose of 15 mg/kg every 24 h is appropriate for adult horses. Clinicians should be aware that total BA concentrations will increase and remain elevated for up to 24 h post‐administration of UDCA.

## 13 Effects of Nebulized Alpha‐2‐Macroglobulin in Adult, Asthmatic Horses

### 13.1 **Rachel Pfeifle** ^1^; Londa Berghaus^2^, MS, PhD; Michelle Coleman^2^, DVM, PhD, DACVIM (LAIM); Kelsey Hart^2^, DVM, PhD, DACVIM (LAIM)

#### 13.1.1 ^1^Clinical Assistant Professor, University of Georgia; ^2^University of Georgia

**BACKGROUND:** Given the broad anti‐inflammatory actions of alpha‐2‐macroglobulin (A2M) in other body systems, investigation into the safety and clinical effects of nebulized A2M in horses as a potential adjunctive management approach for equine asthma warrants consideration.

**OBJECTIVES/HYPOTHESIS:** To evaluate the effects of nebulized A2M on clinical signs, lower respiratory tract inflammation, and pulmonary and systemic cytokine concentrations in asthmatic horses. It was hypothesized that the effects of A2M would not differ from nebulized saline.

**ANIMALS:** Thirteen university‐owned adult, asthmatic horses

**METHODS:** Enrolled horses were randomly divided into two groups. The A2M group received nebulized A2M suspension every 48 h for 6 doses, while the SALINE group received nebulized saline at matching timepoints. Vital parameters and a 23‐point respiratory score were assessed by a blinded veterinarian before each treatment and 24 h after the last treatment. Rebreathing examinations, bronchoalveolar lavage fluid (BALF) for cytology and cytokine analysis, and blood for cytokine concentration were collected before the first and 24 h after the last treatment.

**RESULTS:** There were no significant differences in clinical respiratory scores in the A2M group throughout the study, while the SALINE group had increased scores on days 2, 6, and 8. BALF inflammatory cell percentages and cytokine concentrations in both BALF and serum showed no significant differences between groups at any time.

**CONCLUSIONS AND CLINICAL IMPORTANCE:** Nebulized A2M was well tolerated and did not exacerbate clinical signs in horses with mild asthma. Further investigation into dosing regimens and effects on more severe disease is warranted.

## 14 FOOD ANIMAL INTERNAL MEDICINE

## 15 Assessing the Effectiveness of an Evidence‐Based Algorithm for Antimicrobial Treatment in Neonatal Calf Diarrhea

### 15.1 **Luiza Zakia** ^1^; Mary Brander^2^; Diego Gomez^2^; Peter Constable^3^; Stephen LeBlanc^4^; David Renaud^4^

#### 15.1.1 ^1^Assistant Professor, University of Guelph; ^2^University of Guelph; ^3^College of Veterinary Medicine, University of Illinois; ^4^Department of Population Medicine, Ontario Veterinary College, University of Guelph

**BACKGROUND:** Diarrhea is a major cause of mortality in calves, with antimicrobial drugs (AMD) being used in many cases. However, few guidelines for AMD use in calf diarrhea have been developed.

**OBJECTIVES:** To evaluate the effectiveness of an evidence‐based algorithm (EBA) for antimicrobial treatment of calf diarrhea.

**ANIMALS:** 106 neonatal calves.

**METHODS:** A blinded, non‐inferiority, randomized controlled trial compared two treatments:

1. An EBA, in which calves were treated with AMD (trimethoprim‐sulfadoxine 16 mg/kg) only if they showed ≥ 2 of the following abnormalities:
  - Rectal temperature > 38.8°C after anti‐inflammatory medication (based on cut‐off identified in a prior study)
  - Inability to stand
  - Absent suckle reflex
  - Sunken eyes
  - Scleral injection
2. A control group (CG), where all calves received AMD (trimethoprim‐sulfadoxine) at diarrhea onset

All calves received enteral fluids for the duration of the diarrhea and meloxicam (0.5 mg/kg) at the onset of disease. Cox survival analysis and logistic regression models assessed diarrhea duration, survival, and the need for rescue treatment (i.e., additional AMD therapy).

**RESULTS:** In total, 40% (21/52) of the calves in the EBA group required AMD. Diarrhea duration was not different between groups (CG vs. EBA HR 0.78; 95% CI 0.52–1.16; *p* = 0.22). Control calves had a trend toward higher mortality (OR 3.91; 95% CI 0.90–24.03; *p* = 0.07) and more rescue treatments (OR 6.34; 95% CI 1.28–62.06; *p* = 0.02) compared with EBA calves.

**CONCLUSIONS AND CLINICAL IMPORTANCE:** The EBA did not affect diarrhea duration compared with the CG. However, the EBA trend for lower mortality risk suggests it may improve calf outcomes while reducing AMD use.

## 16 Impact of Three Colostrum Replacement Strategies on Immunoglobulin G Absorption and Growth in Beef Calves

### 16.1 **Lisa Gamsjäger** ^1^; Manuel Chamorro^2^; Danielle Mzyk^3^; Derek Foster^3^; Hakeem Jenkins^3^; Siena Mitman^3^

#### 16.1.1 ^1^Assistant Professor, North Carolina State University; ^2^Auburn University; ^3^North Carolina State University

**BACKGROUND:** Evidence‐based colostrum replacement recommendations are lacking for beef calves.

**OBJECTIVES:** To determine the apparent efficiency of absorption (AEA) of immunoglobulin G (IgG) in beef calves receiving complete colostrum replacement and compare the effects of three replacement strategies on AEA, transfer of passive immunity (TPI), and growth.

**ANIMALS:** 45 newborn Angus calves from university herd

**METHODS:** Randomized clinical trial. Calves were separated from their dams at birth and received either 3.2 L (167 g IgG) of colostrum within 3 h (group A), 4.2 L (245 g IgG) over two feedings within 8 h (group B), or 4.8 L (245 g IgG) over three feedings within 14 h (group C). Serum IgG concentration was measured at 0, 6, 12, and 24 h. The AEA, TPI, and average daily gain (ADG) at weaning were compared among groups using ANOVA, Kruskal–Wallis, Fisher's Exact, or *χ* ^2^ tests, as appropriate.

**RESULTS:** The median AEA was higher in group A (28.5%), compared with groups B (20.7%, *p* = 0.047) and C (18.8%; *p* = 0.005). Median serum IgG concentrations at 24 h were similar at 20.2 g/L (range 6.4–29.4 g/L), 21.2 g/L (range 15.4–32.6 g/L), and 20.1 g/L (range 14.0–27.6 g/L) in groups A, B, and C, respectively (*p* = 0.65). The proportion of calves with IgG concentrations < 10 g/L was small and comparable among study groups (7%, 0%, 0%, *p* = 0.99). There were no significant differences in ADG (*p* = 0.17).

**CONCLUSIONS AND CLINICAL IMPORTANCE:** Early intervention with any of the strategies evaluated was effective in preventing IgG concentrations < 10 g/L, but did not ensure IgG concentrations > 24 g/L.

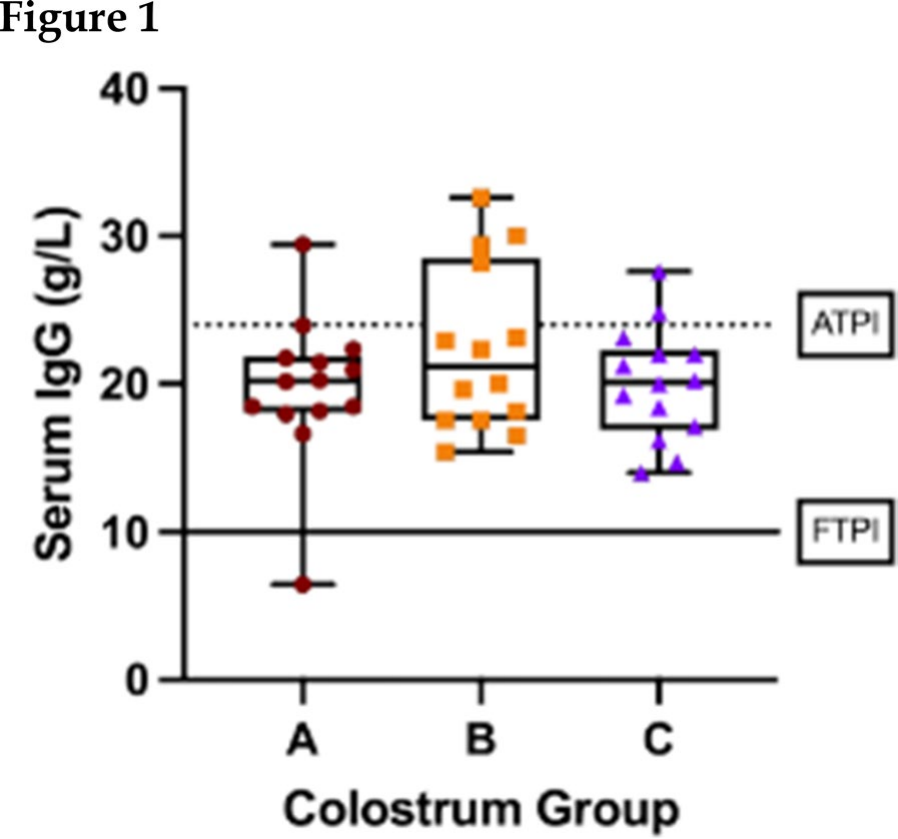

Serum immunoglobulin G (IgG) concentrations in 1‐day‐old beef calves that received 1 of 3 different colostrum replacements. Calves in group A received 3.2 L (167 g IgG) of colostrum within 3 h; calves in group B received 4.2 L (245 g IgG) over 2 feedings within 8 h, and calves in group C received 4.8 L (245 g IgG) over 3 feedings within 14 h. Mean serum concentrations were not significantly different (*p* = 0.65). The solid line depicts the threshold for failed transfer of passive immunity (FTPI, < 10 g/L), and the dashed line depicts the threshold for adequate transfer of passive immunity (ATPI, ≥ 24 g/L) in beef calves.

## 17 Pharmacokinetics of Phenazopyridine in Healthy Goats at Two Different Dosing Regimens

### 17.1 **Jennifer Halleran** ^1^; Sara Fitzgerald^2^; Derek Foster^1^, DVM, PhD, DACVIM; Siena Mitman^2^, DVM, MS; Danielle Mzyk^2^, DVM, PhD; Laura Neumann^2^, MS; Madelyn Schwartz^2^; Dileydis Soto Montes^2^

#### 17.1.1 ^1^Assistant Professor in Ruminant Medicine, North Carolina State University; ^2^North Carolina State University

**BACKGROUND:** Obstructive urolithiasis is one of the most frequent small ruminant emergencies. Because of the possibility of renal compromise, nonsteroidal anti‐inflammatories are sometimes contraindicated for analgesia. Phenazopyridine, a urinary bladder analgesic, may be an alternative.

**HYPOTHESIS/OBJECTIVE:** The objective was to investigate the pharmacokinetics of orally administered phenazopyridine. We hypothesized that the phenazopyridine concentration in the plasma and urine would be increased in the 8 mg/kg every 12‐h dosing regimen when compared with 4 mg/kg every 8 h.

**ANIMALS:** Six male intact Boer goats were used (8–9 months old). They were deemed healthy based on physical examination and blood work.

**METHODS:** The goats were administered 4 mg/kg of phenazopyridine orally every 8 h; blood and urine were collected. After a 7‐day washout period, the goats were orally dosed with 8 mg/kg of phenazopyridine every 12 h. Blood and urine were collected at predetermined time points. Phenazopyridine concentrations were assessed by high‐performance liquid chromatography with UV detection.

**RESULTS:** All of the goats did well during the study. There was no phenazopyridine detected in the urine. In the plasma, phenazopyridine was only detected at 8 h (4 mg/kg dosing group) and at 12 h (8 mg/kg dosing group).

**CONCLUSIONS AND CLINICAL IMPORTANCE:** The pharmacokinetics of phenazopyridine has been elucidated, but phenazopyridine is not well absorbed or excreted in the urine. There appear to be no adverse effects, but the efficacy remains unclear. The metabolites of phenazopyridine may be responsible for any analgesic effect, but this still needs to be characterized.

## 18 NEUROLOGY

## 19 Veterinary Neurologists' Approach to French Bulldogs with Spinal Cord Disease and Brachycephalic Obstructive Airway Syndrome

### 19.1 **Sheila Carrera‐Justiz** ^1^; Elizabeth Rozanski^2^; Christine Rutter^3^

#### 19.1.1 ^1^Clinical Associate Professor, College of Veterinary Medicine, University of Florida; ^2^Tufts University; ^3^Texas A&M University

**BACKGROUND:** French bulldogs (FRBL) are commonly affected with spinal cord diseases (SCD) that require imaging and often surgical decompression/stabilization. FRBL are similarly often affected with brachycephalic obstructive airway syndrome (BOAS). Clinical signs of BOAS may be worsened by stress/anxiety or pain, which commonly affect dogs with SCD. Additionally, recovery from general anesthesia associated with imaging and surgery may precipitate an airway crisis.

**HYPOTHESIS/OBJECTIVES:** The objective was to evaluate neurologists' and neurology residents' views on FRBL with BOAS.

**METHODS:** Neurology listserve members were invited to complete a survey. Participants consented to participate. The survey was exempt from review by the local IRB. Descriptive statistics were used.

**RESULTS:** Exactly 157/579 people responded to the survey for a response rate of 27.1%. The majority (123; 78%) were diplomates, with the remainder residents (27; 17%) and residency‐training (7; 5%). Ninety‐six percent (151/157) have seen an increased number of FRBL in their caseload, with 85% having seen FRBL having airway crises both pre and post imaging/surgery. For the FRBL with severe BOAS signs, 98 (62%) would recommend cooling and trying to avoid airway surgery, 33% (52) would recommend urgent airway surgery (staphylectomy), and 7 (4%) would perform a temporary palatopexy.

**CONCLUSIONS AND CLINICAL IMPORTANCE:** BOAS in FRBL is common and challenging. Consideration should be given to whether training neurologists to perform palliative airway surgery may be warranted.

## 20 Outcome, Complications, and Risk Factors for Perioperative Mortality in Dogs Undergoing Caudal Fossa Surgery

### 20.1 **Adrien Dupanloup** ^1^; Benjamin Shih^2^; Vishal Murthy^2^; Ji‐Hey Lim^2^

#### 20.1.1 ^1^Assistant Professor, Neurology and Neurosurgery, University of California–Davis; ^2^University of California–Davis

**BACKGROUND:** Surgery of the caudal fossa presents distinctive perioperative risks, making the identification of mortality risk factors critical to improving outcomes.

**OBJECTIVES:** To characterize the survival outcomes and perioperative complications in dogs undergoing caudal fossa surgery, and to identify risk factors of early postoperative mortality.

**ANIMALS:** 55 dogs.

**METHODS:** Retrospective study (1995–2024). Data collected included age, weight, histopathologic diagnosis, duration of clinical signs and corticosteroid treatment before surgery, length of anesthesia, and perioperative adverse events.

**RESULTS:** Of 55 dogs, 4 (7.2%) were euthanized intraoperatively, 4 (7.2%) were euthanized before recovery, and 8 (14.5%) died before discharge. On univariate analysis, dogs that survived to discharge (*n* = 39, 70.9%) were significantly younger [median (range): 6 years (1–13)] and had significantly shorter durations of corticosteroid treatment prior to surgery [5 days (0–96)] compared with dogs that died before discharge [9 years (1–16), *p* = 0.014; 22 days (0–74), *p* = 0.007]. Weight (*p* = 0.55), duration of clinical signs (*p* = 0.93), and length of anesthesia (*p* = 0.23) were not associated with survival. On multivariate analysis, odds of survival to discharge decreased with each additional week of corticosteroid treatment pre‐surgery [OR (95% CI): 0.77 (0.57–0.97)] and with each year of age [0.76 (0.60–0.93)]. Pneumonia developed in 7/8 (87.5%) dogs that died before discharge and 5/39 (12.8%) dogs that survived. All dogs that did not survive to discharge had a neoplastic mass. All dogs undergoing subsequent surgery (6/55, 10.9%) survived.

**CONCLUSION:** Survival to discharge after caudal fossa surgery was significantly associated with younger age and shorter corticosteroid treatment duration before surgery.

## 21 Questionnaire‐Based Study of the Correlation Between Sleep and Pain in Cavalier King Charles Spaniels

### 21.1 **Rell Parker** ^1^; Allie Sherman^2^; Mindy Quigley^2^, MA; John Rossmeisl^3^, DVM, MS, DACVIM (SAIM and Neurology); Stephen Werre^4^, PhD

#### 21.1.1 ^1^Assistant Professor, Virginia‐Maryland College of Veterinary Medicine; ^2^Small Animal Clinical Sciences, Virginia‐Maryland College of Veterinary Medicine; ^3^Dr. and Mrs. Dorsey Taylor Mahin Professor of Neurology and Neurosurgery, Veterinary and Comparative Neuro‐Oncology Laboratory, Virginia‐Maryland College of Veterinary Medicine; ^4^Study Design and Statistical Analysis Lab, Virginia‐Maryland College of Veterinary Medicine

**BACKGROUND:** The correlation between sleep and chronic pain in dogs is poorly understood. In people, poor sleep is correlated with chronic pain. It has previously been reported that cavalier King Charles spaniels (CKCS) with caudal occipital malformation may have sleep disturbances.

**HYPOTHESIS/OBJECTIVES:** We hypothesized that CKCS with neuropathic pain (NeP) would have an increased sleep and nighttime restlessness evaluation score (SNoRE3.0) and a worse quality of life (QOL).

**ANIMALS:** 68 CKCS dogs.

**METHODS:** Owners of CKCS were recruited to complete a REDCap survey that was available on the institutional website. It collected demographic information, the SNoRE3.0 sleep survey, and a NeP neuropathic pain questionnaire including a QOL score.

**RESULTS:** The average age of CKCS was 5.0 years. There were 23 male castrated, 23 female spayed, 13 male intact, and 9 female intact dogs. The median SNoRE3.0 score was 12 (range 0–35). The median NeP score was 0.83 (range 0–2.6). The median QOL score was 2 (range 1–5). The Spearman correlation coefficient between the SNoRE3.0 and NeP was 0.381, with *p* = 0.0012. The Spearman correlation coefficient between SNoRE3.0 and QOL was 0.29, with *p* = 0.014. The Spearman correlation coefficient between NeP and QOL was 0.58, with *p* < 0.0001.

**CONCLUSIONS AND CLINICAL IMPORTANCE:** These data indicate there is a positive correlation between owner survey responses regarding increased nighttime restlessness, the presence of neuropathic pain, and a worse QOL. This indicates that sleep may be an important component of neuropathic pain in CKCS.

## 22 Prazosin vs. Tamsulosin for Urinary Retention in Nonambulatory Dogs Following Surgically Treated Intervertebral Disk Herniation

### 22.1 **Han Sun** ^1^; Amy Yanke^2^, DVM, MS, DACVIM (Neurology); Teri Etheredge^3^, PharmD; Theresa Pancotto^4^, DVM, MS, DACVIM (Neurology), CCRP

#### 22.1.1 ^1^Associate Neurologist, Veterinary Neurology Center; ^2^Associate Professor, Neurology & Neurosurgery, Department of Clinical Sciences, College of Veterinary Medicine, Auburn University; ^3^Director of Pharmacy, College of Veterinary Medicine, Auburn University; ^4^Veterinary Neurologist, Specialists in Companion Animal Neurology

**BACKGROUND:** Nonambulatory dogs with thoracolumbar spinal cord injuries frequently develop urinary retention due to increased urethral resistance. This is often pharmacologically addressed with alpha‐adrenergic receptor blockers (e.g., prazosin, tamsulosin). The difference in their efficacy is currently unknown.

**HYPOTHESIS/OBJECTIVES:** Our goal was to determine the efficacy of tamsulosin compared with prazosin in managing urinary retention for nonambulatory dogs with intervertebral disk herniations. We hypothesized that tamsulosin would be better than prazosin regarding the rate of achieving effective bladder expression or voluntary urination, duration of hospitalization, and the subjective ease of bladder expression before discharge.

**ANIMALS:** Client‐owned dogs presenting for a T3–L3 myelopathy undergoing a hemilaminectomy for treatment of intervertebral disk extrusion.

**METHODS:** This double‐blinded, randomized, positive control clinical trial compared the effectiveness of tamsulosin and prazosin for urinary retention post‐hemilaminectomy. The postoperative timeline to reach a small bladder size (measured by ultrasound) after expression and voluntary urination was compared between groups.

**RESULTS:** Twenty‐nine dogs completed the study. Dogs reached effective bladder expression in 2 days (median) in the tamsulosin group, and 1.5 days with prazosin (*p* = 0.44). Effective voluntary urination was achieved in 3.5 days (median) in the tamsulosin group, and 3 days with prazosin (*p* = 0.16). Furthermore, no significance was found between hours to reach effective bladder expression and voluntary urination after starting medications.

**CONCLUSIONS AND CLINICAL IMPORTANCE:** Tamsulosin is clinically comparable to prazosin in treating urinary retention in nonambulatory dogs post‐hemilaminectomy for intervertebral disk extrusion. Dosed once daily, tamsulosin may be a more convenient option for owners.

## 23 NUTRITION

## 24 Highly Hydrolyzed vs. Novel Protein Diet in the Management of Canine Chronic Inflammatory Enteropathy

### 24.1 **Sally Perea** ^1^; Agostino Buono^2^, DVM, PhD, DACVIM (SAIM); Jeremy Laxalde^3^; Yann Queau^3^; Vincent Biourge^3^, DVM, PhD, DACVIM (Nutrition)

#### 24.1.1 ^1^Certified Veterinary Nutritionist, Royal Canin SAS; ^2^Assistant Professor of Small Animal Internal Medicine, Department of Veterinary Clinical Sciences, Louisiana State University; ^3^Royal Canin SAS

**BACKGROUND:** Fifty to 60% of chronic inflammatory enteropathy (CIE) cases are classified as food responsive. Highly hydrolyzed protein formulas composed of amino acids and low‐molecular‐weight peptides have led to remission in otherwise non‐diet‐responsive dogs.

**HYPOTHESIS:** We hypothesized that response would be greater in dogs fed Royal Canin Ultamino (HP) compared with Select Protein Potato and Rabbit (SP) dry diet.

**ANIMALS:** Client‐owned dogs with a history of CIE were recruited from private referral hospitals.

**METHODS:** Clinical investigators and participants were masked, and dogs were randomized to diet. All patients underwent a baseline diagnostic screening and a 2‐, 4‐, and 12‐week follow up. Clinical response was monitored with the canine chronic enteropathy clinical activity index (CCECAI). Non‐responders were offered the opportunity to try the other treatment diet.

**RESULTS:** Twenty‐two dogs completed the study. CCECAI scores improved compared with baseline in both groups at 2, 4, and 12 weeks (*p* < 0.05). Nine of 11 dogs fed the HP (82%) and 5 of 11 dogs fed the SP (45%) achieved clinical response (CCECAI improved by ≥ 75% or was ≤ 1). Six dogs initially fed the SP went on to respond to the HPF after being given the opportunity to switch diets. There were no statistical differences in CCECAI or response rate between diet groups.

**CONCLUSIONS AND CLINICAL IMPORTANCE:** Both the HP and SP formulas were effective in the management of CIE. Dogs that fail to respond to an initial diet trial should undergo a secondary trial, with consideration of a highly hydrolyzed formula.

## 25 ONCOLOGY

## 26 Clinical Efficacy and Tolerability of Lapatinib in Metastatic Canine Mammary Carcinomas: A Multi‐Center Pilot Study

### 26.1 **Doyun Kim** ^1^; Yongsun Kim^2^, DVM, PhD; Mihyun Choi^2^, DVM, PhD; Ja yeon Ryu^3^, DVM; Dongbin Kim^4^, DVM, MS; Kun‐Woo Kim^5^, DVM, MS; Woo‐Jin Song^6^, DVM, DKCVIM, PhD

#### 26.1.1 ^1^Head Oncologist, Bon Animal Medical Center; ^2^President, Bon Animal Medical Center; ^3^Vice President, Internal Medicine, Bon Animal Medical Center; ^4^President, Oncology, Soo Animal Medical Center; ^5^President, Oncology, FM Animal Medical Center; ^6^Professor, Internal Medicine, Jeju National University

**BACKGROUND:** Metastatic mammary carcinomas in dogs are aggressive malignancies with poor survival and limited treatments. Lapatinib, a tyrosine kinase inhibitor targeting HER2, has shown promise in human oncology. Its efficacy in canine metastatic mammary carcinomas requires further study.

**OBJECTIVE:** Evaluate lapatinib's efficacy and tolerability in dogs with metastatic mammary carcinomas, focusing on HER2 expression as a therapeutic biomarker.

**ANIMALS:** Ten client‐owned dogs with histologically or cytologically confirmed metastatic mammary carcinomas (grade 2: *n* = 1, grade 3: *n* = 5, inflammatory mammary carcinoma [IMC]: *n* = 4).

**METHODS:** This multi‐center, retrospective pilot study assessed dogs treated with lapatinib (20–27.4 mg/kg daily). HER2 status was determined via immunohistochemistry (scored 0–3+). Tumor response (RECIST), clinical benefit (CB: complete response [CR], partial response [PR], or stable disease [SD]), tumor‐specific survival (TSS), and adverse effects (VCOG‐CTCAE v2.0) were evaluated.

**RESULTS:** Overall median TSS was 76 days (range: 14–412). HER2‐positive cases (*n* = 3) had a median TSS of 227 days (range: 52–412), HER2‐negative cases (*n* = 3) had 139 days (range: 100–189), and IMC cases (*n* = 4) had 33.5 days (range: 14–43). Non‐IMC (HER2‐positive, HER2‐negative) cases (*n* = 6) achieved 100% CB with a median CB duration of 102 days (range: 51–412). HER2‐positive cases had longer CB duration (median: 227 days, range: 51–412) vs. HER2‐negative cases (93 days, range: 65–111). One HER2‐positive dog (score 2+) with pulmonary metastasis maintained SD for 227 days, and another (score 3+) achieved CR for 412 days; both remained alive at study closure. IMC cases showed progressive disease. Adverse effects in 7 dogs were mild, mainly grade 1–2 liver enzyme elevations.

**CONCLUSIONS AND CLINICAL IMPORTANCE:** Lapatinib showed efficacy in HER2‐positive metastatic mammary carcinomas, prolonging survival compared with HER2‐negative and IMC cases, with good tolerability. HER2 expression supports its role as a biomarker, warranting further study.

## 27 Prognosis and Associated Risk Factors Following Conservative Management of Dogs With Large Liver Tumors

### 27.1 **Chick Weisse** ^1^; Shana Coffey^2^, DVM; Jonathan Ferrari^3^, VMD, DACVS (SA), ACVS Fellow, Surgical Oncology

#### 27.1.1 ^1^Staff Surgeon, Director of IR Service, Schwarzman Animal Medical Center; ^2^Resident Veterinarian in Surgery, Schwarzman Animal Medical Center; ^3^Head of Surgical Oncology, Schwarzman Animal Medical Center

**BACKGROUND:** Information regarding prognosis for conservatively managed hepatic tumors in dogs is limited.

**OBJECTIVES:** Objectives were to report prognosis and identify prognostic factors in dogs with conservatively managed hepatic tumors.

**ANIMALS:** Client‐owned dogs (*n* = 49) with CT‐characterized hepatic tumors that did not pursue definitive therapy and were deceased at the time of analysis.

**METHODS:** Retrospective, single institutional study. Medical records from 2013 to 2023 were reviewed and data collected included date of diagnosis, sex, weight, bloodwork parameters, presenting complaints, tumor volumes, tumor characteristics, and date of death.

**RESULTS:** Median survival time from date of diagnosis was 337 days (range 2–1308). Dogs with a presenting complaint of weight loss (*p* = 0.02) had a significantly shorter median survival time (128 days) than those without weight loss (420 days). Dogs presenting with abdominal effusion (*p* = 0.03) had a significantly shorter median survival time (227 days) than dogs without effusion (331.5 days). Dogs with multiple hepatic masses (*p* = 0.004) had a significantly shorter median survival time (209.5 days) than dogs with a solitary mass (346 days). AST within 30 days of CT was associated with survival time (*p* = 0.02), as was HCT (*p* = 0.01). Tumor volume/body weight ratio was not statistically related to survival time (*p* = 0.8).

**CONCLUSIONS:** The clinical signs of weight loss, abdominal effusion, and presence of multiple hepatic masses, as well as AST and HCT values can be utilized to guide prognostication for dogs undergoing conservative management for hepatic tumors.

## 28 SMALL ANIMAL INTERNAL MEDICINE

## 29 Demographics, Imaging Findings, Diagnosis, and Outcome for 74 Dogs Undergoing Adrenalectomy for an Incidental Mass

### 29.1 **Audrey Cook** ^1^; Phil Mayhew^2^; Federico Massari^3^; Felipe Lillo^4^; Ameet Singh^5^; Bart Van Goethem^6^; Sebastian Vam Nimwegen^7^

#### 29.1.1 ^1^Professor, Small Animal Internal Medicine, College of Veterinary Medicine, Texas A&M University; ^2^University of California–Davis; ^3^Clinica Veterinaria Nervianese; ^4^Universidad Andrés Bello; ^5^Ontario Veterinary College; ^6^Ghent University; ^7^Utrecht University

**BACKGROUND:** Incidental adrenal masses (In‐AMs) are routinely identified in dogs undergoing abdominal imaging. However, there is little information regarding the likelihood of malignancy and the risks vs. benefits of surgical intervention.

**OBJECTIVES:** To compare findings in dogs with In‐AMs against those with clinical adrenal masses (Cl‐AMs). To identify preoperative indicators of histologic diagnosis for dogs with In‐AMs.

**ANIMALS:** 255 dogs undergoing laparoscopic adrenalectomy.

**METHODS:** Demographics, lesion size, and histologic findings were compared for dogs with incidental vs. clinical masses. Survival to discharge was determined for dogs with In‐AMs.

**RESULTS:** Masses were apparently incidental in 74/255 dogs (29.0%). Ages and body weights were similar to those with Cl‐AMs (126.6 ± 25.7 vs. 122.0 ± 27.3 months [*p* = 0.234], and 15 (3.1–96) vs. 11 (3.0–65) kg [*p* = 0.178], respectively). Malignancy rates were similar (56.7% vs. 62.2% [*p* = 0.463]), although In‐AMs were more likely to be pheochromocytomas (34.3% vs. 12.2%; OR 3.76 [1.933–7.42]; *p* = 0.0002). Maximal mass dimension (MMD) was similar for both groups (2.5 [0.9–14] vs. 2.5 [1.3–6.7] cm; *p* = 0.832). Twenty In‐AMs had MMD ≤ 2 cm, 8 of which were noted to have vascular invasion on imaging. However, 6/11 small, noninvasive In‐AMs were malignant (3 pheochromocytomas, 3 carcinomas). Three dogs died from surgical complications.

**CONCLUSION:** These findings suggest that the majority of In‐AMs are malignant, and that size is not predictive of histopathologic findings. Short‐term outcomes following elective laparoscopic adrenalectomy are generally good. Previous recommendations to simply monitor noninvasive In‐AMs with MMD ≤ 2 cm should be reconsidered.

## 30 Risk Factors Against Treating Cats with Diabetes Mellitus and ≤ 30‐day Survival After Starting Anti‐Hyperglycaemic Therapy

### 30.1 **Oliver Waite** ^1^; Dan O'Neill^2^; Emma Wright^2^; Rosanne Jepson^2^; Ruth Gostelow^2^

#### 30.1.1 ^1^Senior Lecturer in Small Animal Medicine, The Animal Hospital, Murdoch University; ^2^Royal Veterinary College

**INTRODUCTION:** Diabetes mellitus (DM) in cats imposes significant caregiver burdens, with 20% of all diabetic cats reportedly dead ≤ 30 days after diagnosis. Risk factors for cats not receiving anti‐hyperglycemic therapy (AHT) are unreported. Studies evaluating early mortality in cats with DM who receive AHT are limited.

**OBJECTIVES/HYPOTHESIS:** Report risk factors for diabetic cats failing to receive AHT, and analyze factors associated with mortality ≤ 30 days following DM diagnosis in cats starting AHT.

**ANIMALS:** 375 cats under United Kingdom primary veterinary care with a first lifetime diagnosis of DM in 2019.

**METHODS:** Anonymized retrospective cohort study using electronic health records from VetCompass. Multivariable logistic regression and Cox proportional hazards models were performed, including risk factors (*p* < 0.2) associated with cats not receiving AHT and ≤ 30‐day mortality when receiving AHT, respectively.

**RESULTS:** Exactly 65/375 (17.3%) diabetic cats did not receive AHT. In cats that started AHT (*n* = 310), estimated ≤ 30‐day mortality was 10.0% (95% CI 7.1–13.8). Cats ≥ 15.0 years had significantly increased odds of not receiving AHT (OR 2.6 [95% CI 1.1–6.0], *p* = 0.024) (Table 1) and also increased ≤ 30‐day mortality hazard after starting AHT (HR 3.5 [95% CI 1.2–10.1], *p* = 0.019) (Table 2). Presenting clinical signs and co‐morbidities were not associated with ≤ 30‐day mortality (*p* > 0.05).

**CONCLUSIONS AND CLINICAL IMPORTANCE:** In this population, almost 20% of diabetic cats were untreated, and greater age was a risk factor for failure to receive AHT and increased ≤ 30‐day mortality. These results provide benchmarking information to assess whether outcomes change following the introduction of sodium‐glucose cotransporter‐2 inhibitors as treatments for feline DM.

**Table:** TABLE 1 Final multivariable logistic regression model for failure to receive AHT among cats with a prevalent (2019) diagnosis of diabetes mellitus (DM) in the VetCompass™ database, whilst under UK primary veterinary care practice in 2019

Variable	Category	Diabetes mellitus cases (2019) no. (%)	Odds ratio	95% confidence interval	*p*
Age (years)	**< 15.0**	**309 (82.4)**	**Base**		
	≥ 15.0	66 (17.6)	2.6	1.1–6.0	0.024*
Blood glucose concentration at diagnosis (mmol/L)	—	225 (60.0)	1.1	1.0–1.2	0.216
Evidence of being overweight ≤ 1 year before diabetes mellitus diagnosis	**No evidence of being overweight**	**297 (79.2)**	**Base**		
	Evidence of being overweight	78 (20.8)	0.7	0.2–1.8	0.426
Weight loss	**Not recorded**	154 (41.1)	Base		
	Recorded	221 (58.9)	0.7	0.4–1.7	0.509

**Table:** TABLE 2 Final multivariable Cox regression model for early mortality (≤ 30 days) among cats with a prevalent (2019) diagnosis of diabetes mellitus (DM) in the VetCompass database, while under UK primary veterinary care practice in 2019, following the start of AHT

Variable	Category	Diabetic cats treated with anti‐hyperglycemic therapy no. (%)	Hazard ratio	95% confidence interval	*p*
Age (years)	**< 15.0**	**259 (83.5)**	**Base**		
	≥ 15.0	**51 (16.5)**	3.5	1.2–10.1	0.019*
Adult bodyweight at diagnosis (kg)	—	275 (88.7)	1.2	0.8–1.7	0.439
Evidence of obesity within 1 year of diabetes mellitus diagnosis	**No evidence of obesity reported**	**242 (78.1)**	**Base**		
	Evidence of obesity reported	68 (21.9)	3.0	1.0–9.1	0.058
Blood glucose concentration at diagnosis (mmol/L)	**< 25.0**	**97 (31.3)**	**Base**		
	≥ 25.0	**88 (28.4)**	0.4	0.1–1.4	0.164
Comorbidity	**Not present**	274 (88.4)	**Base**		
	Present	36 (11.6)	0.0	0.0–0.0	0.982
Diarrhea reported as a presenting clinical sign within 4 weeks of diabetes mellitus diagnosis	**Diarrhea not present**	**283 (91.3)**	**Base**		
	Diarrhea present	27 (8.7)	2.5	0.7–9.1	0.172

## 31 Clinical Experience with Fuzapladib Sodium Treatment in Dogs with Complicated Acute Pancreatitis

### 31.1 **Sue Yee Lim** ^1^; Jessica Herman^2^, DVM; Abby Ostronic^2^; Kellyn McNulty^3^, DVM, DACVIM (SAIM); Jörg Steiner^4^, MedVet, DrMedVet, PhD, DACVIM, DECVIM‐CA, AGAF

#### 31.1.1 ^1^Assistant Professor, Gastrointestinal Laboratory, Small Animal Internal Medicine, Texas A&M University; ^2^Resident, Small Animal Internal Medicine, Department of Small Animal Clinical Sciences, Texas A&M University; ^3^Department of Small Animal Clinical Sciences, Texas A&M University; ^4^Regents Professor, University Distinguished Professor, Dr. Mark Morris Chair in Small Animal Gastroenterology and Nutrition Director, Gastrointestinal Laboratory, Small Animal Internal Medicine Texas A&M University

**BACKGROUND:** Treatment with fuzapladib sodium for dogs with complicated acute pancreatitis (cAP) and comorbidities has not been reported to date.

**OBJECTIVE:** To describe the comorbidities, survival, and duration of hospitalization in dogs with cAP treated with fuzapladib sodium.

**ANIMALS:** 17 client‐owned dogs with cAP that were treated with fuzapladib sodium

**METHODS:** Retrospective case series. Cases were identified by review of hospital medical records. Signalment, comorbidities, clinicopathology, survival to discharge, and duration of hospitalization were documented.

**RESULTS:** Median age and body weight were 9 years (range 2–16) and 8 kg (2–37). When diagnosed with AP, 94% (16/17) of dogs had various comorbidities including active inflammation/infection (*n* = 8), recent abdominal surgery (*n* = 5), coagulopathy (*n* = 6), and endocrinopathy (*n* = 3). Dogs with endocrinopathies presented with diabetic ketoacidosis, diabetes, and pituitary‐dependent hypercortisolism (*n* = 1 each). Fuzapladib treatment was started at a median of 2 days (1–5) after the diagnosis of cAP. On day 1 of fuzapladib treatment, the median leukocyte count was 16,500/μL (5,800–84,000), the median neutrophil count was 13,695/μL (3,654–62,937), and the median band count was 420/μL (0–10,101). No severe adverse events related to fuzapladib treatment were noted. 76% (13/17) survived to discharge with a median duration of hospitalization of 4 days (2–23). The remaining 24% (4/17) of dogs were euthanized due to disease progression.

**CONCLUSIONS AND CLINICAL IMPORTANCE:** Fuzapladib sodium appeared beneficial for treating cAP in this group of dogs with various comorbidities and resulted in favorable outcomes as evidenced by a high survival to discharge and a relatively short duration of hospitalization.

## 32 Transcriptomic Interrogation of the Duodenal Mucosa of Dogs With Chronic Enteropathy

### 32.1 **Alison Manchester** ^1^; Lyndah Chow^2^, PhD; Michael Lappin^2^, DVM, PhD, DACVIM (SAIM); Steve Dow^2^, DVM, PhD, DACVIM (SAIM)

#### 32.1.1 ^1^Postdoctoral Fellow, Colorado State University; ^2^Colorado State University

**BACKGROUND:** Canine chronic enteropathy (CE) is a complex and incompletely understood syndrome. Bulk transcriptomic (RNA‐seq) analysis offers characterization of thousands of genes simultaneously. Application of this technique to intestinal biopsies enables determination of the molecular signatures of enteric diseases.

**HYPOTHESIS/OBJECTIVES:** To use RNA‐seq to define canine duodenal gene expression and compare transcriptomic programs in health and disease.

**ANIMALS:** 4 healthy (3 research colony, 1 client‐owned) and 11 client‐owned dogs with CE and serum albumin concentrations > 2.5 g/dL.

**METHODS:** Descriptive case‐control study. Duodenal biopsies were collected during GI endoscopy and RNA was isolated using a commercial kit. mRNA sequencing was completed. Gene expression in CE samples were compared with healthy using DESeq2 and pathway analysis using GSEA.

**RESULTS:** Transcriptional profiling revealed heterogeneity within both populations. Differential gene expression analysis highlighted 783 significantly upregulated and 1496 down‐regulated genes in the CE group compared with healthy. This included enhanced expression of cytochrome P450 family 1 subfamily A member 1 (CYP1A1), glutathione peroxidase 2, and ATPase phospholipid transporting 10B (ATP10B) and diminished expression of cell migration inducing hyaluronidase 1 (CEMIP) and POU class 2 homeobox associating factor 1 within the CE group. GSEA highlighted enhancement of mitochondrial oxidative phosphorylation and electron transport pathways, with diminished epithelial to mesenchymal transition and cytokine receptor interaction pathways in CE samples.

**CONCLUSIONS AND CLINICAL IMPORTANCE:** RNA sequencing of duodenal tissues provides comprehensive data highlighting molecular contributions to canine CE. This approach will support hypothesis‐based investigations and advance mechanistic understanding of disease.

## 33 Insect‐Based Novel Protein Diet for Dogs With Chronic Enteropathy: A Prospective Study

### 33.1 **Kevin Murtagh** ^1^; Eva Stavroulaki^2^; Miguel Carvalho^3^; Emma O'Neill^4^; Carmel Mooney^4^

#### 33.1.1 ^1^Associate Professor, Internal Medicine, School of Veterinary Medicine, University College Dublin, Dublin, Ireland; ^2^Resident, Internal Medicine, Dick White Referrals Veterinary Specialists; ^3^Internal Medicine, School of Veterinary Medicine, University College Dublin, Dublin, Ireland; ^4^Professor, Internal Medicine, School of Veterinary Medicine, University College Dublin, Dublin, Ireland

**BACKGROUND:** Limited data exist on the efficacy of insect‐based diets in canine chronic enteropathy (CE).

**HYPOTHESIS/OBJECTIVES:** To determine the effect of an insect‐based diet in dogs with CE.

**ANIMALS:** Dogs with CE were prospectively recruited.

**METHODS:** Body weight, 9‐point BCS, 7‐point fecal score, and CIBDAI were assessed at day 0, 14, 30, and 90. Data were assessed for normality using the Shapiro–Wilk test and described as mean (SD) or median (range), as appropriate, and statistical analysis using the Friedman test for repeated measures and Dunn's multiple comparison test was performed.

**RESULTS:** Exactly 25 dogs were recruited; 5 were withdrawn. 14 (70.0%) responded. Median weight for responders was 13.6 (6.25–39.3) kg at baseline, 13.2 (6.67–31.5) kg at day 14, 14.2 (7.1–39.8) kg at day 30, and 13.3 (7–39.3) kg at day 90, with a statistically significant difference found (*p* < 0.05). Post hoc analysis showed a statistically significant difference between baseline and days 30 and 90 (*p* < 0.05). BCS improved from a median of 4 (2–6) at baseline to 5 (4–6) at day 90 (*p* < 0.05). Post hoc analysis revealed significant improvements between baseline and day 30 (*p* < 0.05) with further increases by day 90 (*p* < 0.05). Median fecal scores significantly improved from 6 (4–7) at baseline to 2 (2–4) at days 30 and 90 (*p* < 0.05). Median CIBDAI decreased from 8 (5–14) at baseline to 0 (0–3) at day 90 (*p* < 0.05).

**CONCLUSIONS AND CLINICAL IMPORTANCE:** An insect‐based diet improves clinical outcomes in canine CE, offering an effective treatment option.

## 34 Neutrophilic Inflammatory Enteropathy in Dogs: A Retrospective Descriptive Study

### 34.1 **Kevin Murtagh** ^1^; Pamela Kelly^2^; Rob Shiel^3^; Emma O'Neill^4^; Carmel Mooney^4^

#### 34.1.1 ^1^Associate Professor, Internal Medicine, School of Veterinary Medicine, University College Dublin, Dublin, Ireland; ^2^Assistant Professor, Pathology, School of Veterinary Medicine, University College Dublin, Dublin, Ireland; ^3^Professor, Internal Medicine, School of Veterinary Medicine, School of Veterinary Medicine, Murdoch University, Perth, Australia; ^4^Professor, Internal Medicine, School of Veterinary Medicine, University College Dublin, Dublin, Ireland

**BACKGROUND:** Neutrophilic inflammatory enteropathy is poorly described in dogs.

**HYPOTHESIS/OBJECTIVES:** To describe the clinical and diagnostic findings, outcome, and survival in a population of dogs with histologically confirmed neutrophilic enteropathy.

**ANIMALS:** Client‐owned dogs with histologically confirmed neutrophilic enteropathy.

**METHODS:** Histopathology reports (2015–2024) with neutrophilic small intestinal inflammation were identified. Individual case records were reviewed. Quantitative data were assessed for normality using the Shapiro–Wilk test and described as mean (SD) or median (range) as appropriate.

**RESULTS:** 26 cases were identified, representing 22 breeds with a mean age of 92 (±43.1; 95% CI 74.5–109.3) months and a median weight of 16.2 (5.1–45.3) kg. The most common presenting signs included diarrhea (*n* = 20, 76.9%), vomiting (*n* = 20, 76.9%), weight loss (*n* = 19, 73.1%), anorexia/hyporexia (*n* = 12, 46.2%), and melaena (*n* = 7, 26.9%). Clinicopathological testing identified a mean albumin concentration of 25.5 (±6.1, 95% CI 23.1–28) g/L, with 5 (19.2%) values < 20 g/L and a median cholesterol concentration of 3.5 (1.6–11.2) mmol/L, with 7 (26.9%) values < 2.5 mmol/L. Folate was measured in 22 cases, with a median concentration of 13.85 (3.47–24.1) ng/mL, with 6 (27.3%) values < 8.2 ng/mL. Cobalamin was assessed in 23 cases, with a median concentration of 239 (149–1001) pg/mL, with 6 (26.1%) values < 150 pg/mL. The median hospitalization time was 4 (1–14) days, with 24 dogs surviving to discharge and an overall median survival time of 322 days.

**CONCLUSIONS AND CLINICAL IMPORTANCE:** Neutrophilic enteropathy is an important consideration for dogs presenting with gastrointestinal signs, including protein‐losing enteropathy, and could be associated with a poor prognosis.

## 35 Fecal Microbiota Transplantation as Adjunct Treatment in Dogs With Refractory CE: A Prospective Study

### 35.1 **Linda Toresson** ^1^; Ulrika Ludvigsson^2^; Gunilla Olmedal^2^; Michaela Toni^2^; Josefin Hellgren^2^; Paula Giaretta^3^; Jan Suchodolski^3^

#### 35.1.1 ^1^Postdoctoral Research Associate, Gastrointestinal Laboratory, Texas A&M University, College Station, TX, USA; ^2^Evidensia Specialist Animal Hospital, Helsingborg, Sweden; ^3^Gastrointestinal Laboratory, Texas A&M University, College Station, TX, USA

**BACKGROUND:** Fecal microbiota transplantation (FMT) is promising as adjunct therapy in dogs with chronic enteropathies (CE), but prospective longitudinal studies are lacking.

**OBJECTIVES:** Report fecal and clinical parameters in dogs with refractory CE treated with FMT

**ANIMALS:** 41 dogs with refractory CE.

**METHODS:** Prospective observational study. CE dogs were treated with rectal FMT as adjunct therapy on 2–3 occasions. Fecal samples were collected, and the canine inflammatory bowel disease activity index (CIBDAI) was calculated for 3 months after inclusion. A positive response to FMT was defined as a decrease of CIBDAI 28–35 days after inclusion, without changes to treatment or diet, or being able to decrease concurrent medication. Dysbiosis index (DI) was used for fecal microbiota assessment.

**RESULTS:** 29/41 dogs responded to FMT, of which 8 dogs had a response lasting < 30 days post FMT. Corticosteroids could be tapered in 14 dogs. Baseline CIBDAI (median (range)) was 5 (2–13) in responders, which decreased to 2 (1–5) 28–35 days after inclusion, without further increase at day 88–95 (*p* < 0.001, mixed‐effect analysis with multiple comparisons). Baseline CIBDAI in non‐responders was 5.5 (2–9) (*p* = 0.42 vs. responders, Mann‐Whitney test) without improvement at follow‐up (*p* = 0.67, paired t‐test). Good responders had lower DI at inclusion (0.1 (−7 to 8.7)) vs. non‐ and short‐lasting responders (4.2 (−4.6 to 8.6) *p* = 0.04, unpaired *t*‐test).

**CONCLUSIONS AND CLINICAL IMPORTANCE:** FMT can be effective and potentially corticoid‐sparing adjunct treatment in refractory CE dogs. Dogs with severe dysbiosis may respond less favorably to FMT.

## 36 Treatment With Bile Acid Sequestrants in Dogs with Refractory Chronic Enteropathies

### 36.1 **Linda Toresson** ^1^; Amanda Blake^2^; Ulrika Ludvigsson^3^; Gunilla Olmedal^3^; Paula Giaretta^2^; Chi‐Hsuan Sung^2^; M. Tolbert^2^; Jan Suchodolski^2^

#### 36.1.1 ^1^Postdoctoral Research Associate, Gastrointestinal Laboratory, Texas A&M University, College Station, TX, USA; ^2^Gastrointestinal Laboratory, Texas A&M University, College Station, TX, USA; ^3^Evidensia Specialist Animal Hospital, Helsingborg, Sweden

**BACKGROUND:** A poor response to standard therapy is common in dogs with chronic enteropathy (CE) and is sometimes associated with euthanasia. A recent report suggests that some of these dogs with refractory CE respond to bile acid sequestrants (BAS).

**OBJECTIVES:** Characterize fecal and clinical parameters in dogs with refractory CE responding vs. not responding to BAS

**ANIMALS:** 24 dogs with refractory CE and 18 healthy dogs.

**METHODS:** Retrospective case series. CE dogs were treated with BAS (cholestyramine, colestipole, or colesevelam) as adjunctive therapy after collection of a fecal sample. Canine inflammatory bowel disease activity index (CIBDAI) was compared at baseline and follow‐up. Response to BAS was defined as a decrease of CIBDAI within 35 days. Fecal samples were analyzed with the dysbiosis index (DI) and LC‐MS/MS for BAs.

**RESULTS:** Exactly 16/24 CE dogs responded favorably to BAS. CIBDAI decreased significantly with (median, (range)) 3 (1–10) units in BAS responders (*p* < 0.001), but not in non‐responders (*p* = 0.13; Wilcoxon matched‐pairs signed rank test for both comparisons). BAS responders had increased amounts and percentages of fecal primary BAs vs. non‐responders and healthy dogs (*p* < 0.001 and *p* < 0.001, Kruskal–Wallis test). The DI was higher and *Peptacetobacter hiranonis* abundance lower in responders vs. non‐responders (*p* = 0.02 and *p* = 0.05, Mann–Whitney test).

**CONCLUSIONS AND CLINICAL IMPORTANCE:** A subgroup of dogs with refractory CE responds favorably to BAS. The responders were characterized by increased fecal primary BAs and DI, and decreased abundance of *P. hiranonis*, versus BAS non‐responders.

## 37 Evaluating the Impact of Preparation Conditions on Bacterial Viability in Canine Fecal Microbiota Transplant Capsules

### 37.1 **Meg Nakazawa** ^1^; Shino Yoshida^2^; Keiko Kato^2^; Christopher Reis^3^; Trevor Alexander^3^; Claire Burbick^3^; Yoko Ambrosini^2^, DVM, MPVM, PhD, DACVIM (SAIM)

#### 37.1.1 ^1^Postdoctoral Researcher, Washington State University; ^2^Department of Veterinary Clinical Sciences, Washington State University; ^3^Washington Animal Disease Diagnostic Laboratory, Washington State University

**BACKGROUND:** Fecal microbiota transplantation (FMT) restores gut microbiota by transferring stool from healthy donors to patients with gastrointestinal diseases. In humans and dogs, FMT has been shown to benefit acute diarrhea, chronic enteropathy, and *Clostridium difficile*‐associated diarrhea. However, research on fecal preparation methods, particularly in veterinary medicine, remains limited.

**OBJECTIVES:** This study aimed to evaluate the effects of freeze‐thaw cycles, oxygen exposure, and gastric acidity on bacterial viability in canine feces.

**METHODS:** Mock fecal samples were prepared by sterilizing donor feces and adding a bacterial mixture (*E. coli*, *E. faecium*, *C. perfringens*, and *B. bifidum*; 7–8 log CFU/g). Glycerol and water were incorporated, and capsules were created using the Tend‐Health CAD device. Bacterial viability was assessed at 0, 7, and 90 days post‐encapsulation.

**RESULTS:** Freezing significantly reduced bacterial viability, particularly for facultative anaerobes such as *E. coli* (day 14: 3.50 log CFU/g; day 90: 3.81 log CFU/g decrease, *p* < 0.001) and *E. faecium* (day 14: 0.63 log CFU/g; day 90: 0.65 log CFU/g decrease, *p* < 0.001). Anaerobic bacteria were undetectable after 7 days. Oxygen exposure and gastric acidity had minimal effects on bacterial survival.

**CONCLUSIONS:** Freezing significantly impacts bacterial viability, while oxygen exposure does not, eliminating the need for an anaerobic chamber during encapsulation. Acid‐resistant capsules effectively withstand gastric pH, providing a practical method for producing oral FMT capsules that maintain bacterial integrity, offering a promising approach to treat gastrointestinal diseases in dogs and cats.

## 38 Outcomes and Long‐Term Survival in Animals Treated for Hepatic Arteriovenous Malformations (HAVM)

### 38.1 **Chick Weisse** ^1^; Allyson Berent^2^; Robert Rosen^3^; Anjile An^4^

#### 38.1.1 ^1^Staff Surgeon, Director of IR Service, Animal Medical Center; ^2^Animal Medical Center; ^3^Lenox Hill; ^4^Weill Cornell

**INTRODUCTION:** Hepatic arteriovenous malformations (HAVM) are complex congenital vascular anomalies associated with varying degrees of liver dysfunction, hepatoencephalopathy, ascites, and failure to thrive. Due to high morbidity associated with hepatic lobectomy, alternative treatments including medical management, transarterial glue embolization (TAE), and dominant outflow vein (DOV) occlusion have been pursued. The goal of this study was to compare long‐term outcomes in terms of patient survival following medical and various surgical techniques.

**MATERIALS AND METHODS:** Medical records from an IR service were reviewed for all HAVM patients treated over a 20‐year period. Information collected included patient signalment, presenting clinical signs, diagnostic imaging and biochemical findings, medical, surgical/interventional managements pursued with associated complications, and patient outcomes including medication requirements and median survival times (MST). Patients were excluded from the study if final follow‐up information was less than 1 year or not available via contact with the client or referring veterinarian.

**RESULTS:** Exactly 46 patients were identified, including 43 dogs and 3 cats. In dogs, medical management alone in 4 animals yielded a MST of 567 d, while surgery provided a MST of 1278 d. While surgical complications and MST were not significantly different between the surgical treatment groups, the TAE group was significantly more likely to have remaining HAVM blood flow immediately after treatment (44% vs. 5%) and experience HAVM recurrence (53% vs. 15%), prompting additional treatments compared with the DOV group.

**CONCLUSION:** Patients with HAVM may have improved outcomes following surgical treatment. TAE and DOV occlusion have similar overall outcomes but different recurrence rates.

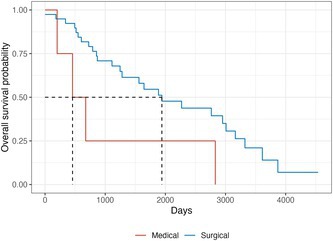

## 39 Infection Prevention and Control Programs at AVMA‐Accredited Veterinary Teaching Hospitals

### 39.1 **Marion Allano** ^1^; Brandy Burgess^2^, DVM, MSc, PhD, DACVIM (LAIM), DACVPM

#### 39.1.1 ^1^Infection Prevention and Control Specialist Advisor, Lecturer in Veterinary Medicine, Université de Montréal; ^2^Not provided

**BACKGROUND:** Biosecurity programs are essential in veterinary teaching hospitals (VTHs). While general recommendations are well‐known, their practical implementation is a challenge.

**HYPOTHESIS/OBJECTIVES:** To describe recent infection and prevention control practices and compare with previous results.

**ANIMALS:** 18 biosecurity experts at 17 AVMA‐accredited VTHs.

**METHODS:** Cross‐sectional study. An online survey was submitted to each institution between September 2023 and December 2024.

**RESULTS:** Outbreaks of nosocomial infections and restricted patient admissions in the past 5 years were reported respectively in 9 and 8 of 15 VTH. Six of 9 VTH reported more than 1 episode: *Salmonella* was the most frequent agent in large animals, and multidrug‐resistant bacteria in small animals. Environmental tests are done routinely at various frequencies in 11 of 16 VTH: *Salmonella*, CRE, MRSA, and MRSP are the most commonly reported bacteria actively tested. A majority of institutions (10/16) routinely test animals to detect contagious agents: most frequently *Salmonella* in equines and carbapenem‐resistant Enterobacteriaceae (CRE) in small animals. In 2 of 15 VTH, the nosocomial infection rates are monitored over time. All respondents reported having a committee that oversees the program, and written policy documents. Just over half (7/13) of the institutions have an antimicrobial stewardship committee.

**CONCLUSIONS AND CLINICAL IMPORTANCE:** In the context of scarce evidence‐based information, sharing local practices is useful. The structure of the programs is evolving, but there is heterogeneity in protocols, data collection and reporting, and surveillance tools. (Comparisons with previous studies are underway, Benedict *JAVMA* 2008.)

## 40 Prolonged Antibody Responses in Dogs and Cats Exposed to COVID‐19 Diagnosed Pet Owners

### 40.1 **Anne Kimmerlein** ^1^; Talon McKee^2^; Philip Bergman^2^; Irina Sokolchik^3^; Christian Leutenegger^3^, DVM, BSc, PhD, FVH

#### 40.1.1 ^1^Epidemiologist, VCA Animal Hospitals, Inc.; ^2^VCA Animal Hospitals, Inc.; ^3^Antech Diagnostics, Inc.

**BACKGROUND:** Dogs and cats can be infected with SARS‐CoV‐2 and develop various clinical presentations. Active surveillance of potentially zoonotic pathogens in pets is required as part of the One Health initiative's aim to study the interaction of pathogens between people, animals, and the environment.

**OBJECTIVES:** To investigate immunoglobulin responses after dog and cat short‐ and long‐term exposure to COVID‐19 diagnosed pet owners.

**ANIMALS:** A total of 1000 serum samples from dogs (*n* = 747) and cats (*n* = 253) owned by 1299 surveyed pet owners in the United States were evaluated.

**METHODS:** Serum samples were analyzed using recombinant nucleocapsid and spike proteins using enzyme‐linked immunosorbent assay (ELISA) tests for IgM and IgG antibodies. Time between exposure and antibody testing was collected from unique pet owner‐reported survey information for individual dogs and cats. The proportion of antibody responses was compared using generalized mixed‐effects logistic regression (GLMER).

**RESULTS:** Immunoglobulin responses were more frequently directed against spike than nucleocapsid proteins in dogs and cats (Table 1). IgG responses were more frequent than IgM responses in both species. The longest time interval between reported COVID‐19 exposure and immunoglobulin detection was 840 days in dogs and 660 days in cats. IgG and IgM had similarly long intervals between exposure and immunoglobulin testing in dogs, but not in cats.

**CONCLUSIONS:** Protracted IgG and IgM responses in US dogs suggest repeated exposure to SARS‐CoV‐2. Differences in husbandry practices, or species‐specific immune systems between dogs and cats may explain the different immunoglobulin responses in our study.

**Table:** TABLE 1 Distribution of immunoglobulin detection and IgG and IgM responses in cats and dogs using ELISA

	Dogs (*n* = 747)	Cats (*n* = 253)
	*N* (%)	*N* (%)
Antibody type		
IgG or IgM	247 (33.1)	68 (26.9)
IgG	225 (30.1)	67 (26.5)
Spike IgG	200 (26.8)	55 (21.7)
NC IgG	79 (10.6)	32 (12.6)
IgM	70 (9.4)	2 (0.8)
Spike IgM	42 (5.6)	2 (0.8)
NC IgM	50 (6.7)	0
Ig type		
IgG	177 (23.7)	66 (26.1)
IgM	22 (2.9)	1 (0.4)
IgG and IgM	48 (6.4)	1 (0.4)

## 41 Comparative Evaluation of Two Canine Vector‐Borne Disease Pathogen PCR Panels

### 41.1 **Christian Leutenegger** ^1^; Michelle Evason^2^; Greg Freeman^2^; Pablo David Jimenez Castro^2^; Christian Savard^2^; Irina Sokolchik^2^; Jeffrey Tereski^2^; Jennifer Wilcox^2^

#### 41.1.1 ^1^VP Research and Development, Antech Diagnostics, Inc., Mars Pet Care Science and Diagnostics, Antech/Mars; ^2^Antech/Mars

**BACKGROUND:** Serology (antibody) testing is typically used for tick‐borne pathogen screening by veterinary clinics and laboratories. With the emerging number, One Health importance, and increased range of vector‐borne disease (VBD) pathogens, broad molecular screening panels that include drug resistance testing may be needed and assist in the determination of clinical management.

**OBJECTIVE:** To compare the performance of two canine real‐time (qPCR) VBD panels for pathogen screening.

**ANIMALS:** A group of 133 canine remnant whole blood samples from the southeastern United States (US) and Texas was collected. Samples were screened for sufficient blood volume for performance analysis and comparative evaluation.

**METHODS:** Results from both qPCR panels were evaluated to determine the frequency of detection for six canine parasites. Frequency calculations included overall parasites detected, total parasite‐positive samples, and co‐infections.

**RESULTS:** A total of 56.4% (75/133) and 54.1% (72/133) parasite pathogens were detected by panels 1 and 2, respectively. Positive samples were described (Table), and for both panels were detected positive in 46.6% (62/133). Co‐infections were detected by panels 1 and 2, in 9 and 7 samples, respectively.

**CLINICAL IMPORTANCE:** These data describe the parasite‐pathogen proportion positive in this cohort. Our performance comparison indicates a high degree of agreement between two commercially available VBD qPCR panels used for pathogen screening in dogs. Due to evolving vector biology and increasing One Health importance in the U.S. and Canada, there is a need to continuously update molecular VBD pathogen panels. Broader qPCR panels that include drug resistance markers may aid timely clinical decision‐making and veterinary antimicrobial stewardship efforts.

**Table:** TABLE 1 Comparative performance of vector‐borne disease (VBD) qPCR panels for pathogen screening in dogs, *n* = 133

	Panel 1: qPCR panel Antech, Mars Petcare Science & Diagnostics	Panel 2: qPCR panel North Carolina State University
Pathogens detected	56.4%	54.1%
*Ehrlichia* spp.	20.3%	19.5%
*Babesia* spp.	21.1%	20.3%
*Babesia gibsoni*	10.5%	9.8%
*Anaplasma phagocytophilum*	9.0%	9.8%
Hemotropic *Mycoplasma*	4.5%	4.5%
*Hepatozoon canis*	3.0%	0.8%
Samples positive	46.6%	46.6%
Co‐infections	14.5%	11.3%

## 42 Relationship Between Urinary Ammonia Excretion and Survival Time in Dogs With Kidney Disease

### 42.1 **Autumn Harris** ^1^; Alexis Cooper^2^; Rebeca Castro^3^; Andrew Specht^2^, DVM, DACVIM (SAIM); Allison Kendall^3^, DVM, MS, DACVIM (SAIM); Shelly Vaden^3^, DVM, PhD, DACVIM (SAIM), Founding Member ACVNU; Kirsten Cooke^2^, DVM, DACVIM (SAIM)

#### 42.1.1 ^1^Associate Professor, Nephrology‐Urology, North Carolina State University; ^2^University of Florida; ^3^North Carolina State University

**BACKGROUND:** Inadequate ammonia excretion drives the development of metabolic acidosis in people with chronic kidney disease (CKD) and is associated with worse clinical outcomes. Limited information exists about ammonia excretion in dogs with CKD.

**HYPOTHESIS/OBJECTIVES:** We hypothesized that impaired urine ammonia‐to‐creatinine ratio (UACR) would be associated with worse clinical outcomes in dogs with stable CKD.

**ANIMALS:** 60 client‐owned dogs with stable IRIS stage II–IV CKD.

**METHODS:** Single institution prospective longitudinal study. Dogs were followed until death or study end (12 months). Survival times were calculated from the date of study inclusion. Univariable Cox regression was used to assess variables associated with survival. Dogs with > 25% change in serum creatinine were classified as having progressive CKD and compared with dogs with stable CKD using the Mann–Whitney test.

**RESULTS:** Impaired ammonia excretion (UACR < 2.0) was associated with the risk of death (hazard ratio 2.814; CI 1.067–8.115), with a significant survival difference between dogs with UACR < 2.0 (189 days) and dogs with UACR > 2.0 (445 days) (*p* = 0.008). Progressive CKD dogs (*n* = 27) had lower ammonia excretion (UACR 1.7; 0.2–5.5) compared with stable CKD dogs (*n* = 23) (UACR 3.2; 0.3–7.7) (*p* = 0.006).

**CONCLUSIONS AND CLINICAL IMPORTANCE:** Dogs with CKD and impaired ammonia excretion (UACR < 2.0) had 2.8 times greater risk of death and shorter survival times compared with dogs with unimpaired ammonia excretion (UACR > 2.0). Dogs with progression of their CKD had significantly lower ammonia excretion at enrollment compared with dogs with stable CKD. UACR may be a useful biomarker for determining alkali therapy use.

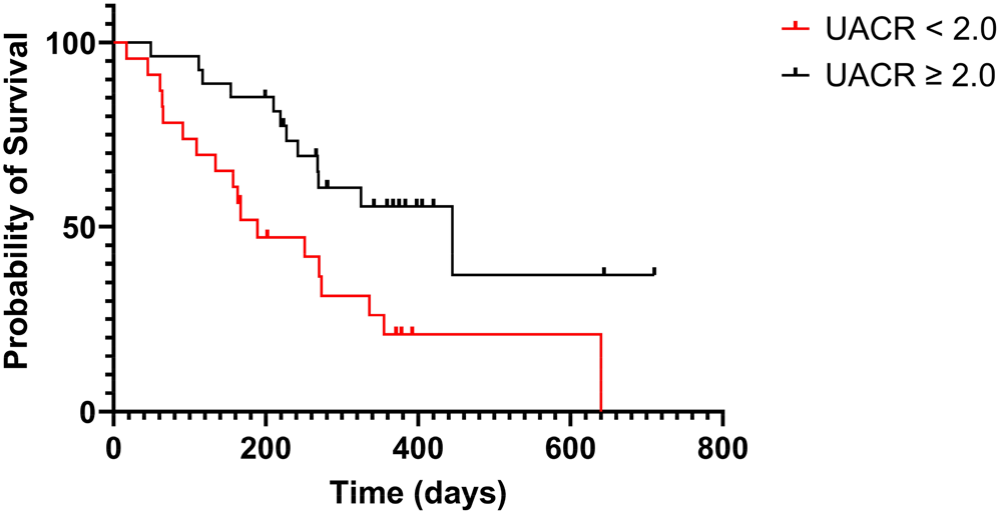

## 43 Prevalence of B‐Lines in Cats With Asthma

### 43.1 **Elizabeth Rozanski** ^1^; Natassja Boham^2^, DVM; Alexia Berg^2^, DVM, DACVECC; Soren Boysen^3^, DVM, DACVECC

#### 43.1.1 ^1^Associate Professor, Tufts University; ^2^Tufts University; ^3^University of Calgary

**BACKGROUND:** Asthma is common in cats and people, with 30% of asthmatic children having B‐lines identified on lung ultrasound (LUS) that correlate with the severity of asthma. Although LUS is often used to document the sonographic presence of hyperechoic vertical artifact, particularly B‐lines, research is lacking on its application in feline asthma.

**HYPOTHESIS/OBJECTIVES:** The objective is to prospectively evaluate if asthmatic cats have B‐lines, and if they correlate to disease severity as assessed by oxygen and hospital admission.

**ANIMALS:** Cats were identified with asthma based on a combination of history, clinical signs, radiographs, and airway cytology.

**METHODS:** The presence/absence of sonographic B‐lines, and when present, their quantification was recorded. Cats were classified as outpatient/inpatient, and for cats receiving oxygen, the duration of therapy was recorded. Descriptive statistics were calculated, and a *t*‐test was used to compare B‐lines with the duration of stay and supplemental oxygen requirements (significance considered *p* ≤ 0.05).

**RESULTS:** Ten cats were included. B‐lines were identified in 4 cats (range 2–5/cat) and absent in 6. Nine cats received oxygen. Seven cats were hospitalized. There was no difference between length of stay and duration of oxygen supplementation in cats with or without B‐lines.

**CONCLUSIONS AND CLINICAL IMPORTANCE:** The presence of B‐lines does not exclude a diagnosis of asthma, although B‐lines were uncommon. Evaluation of a larger number of cats is required for more definitive statements on the predictive value of B‐lines regarding the severity of feline asthma.

